# SARS-CoV-2 Vaccination for Children—An Open Issue

**DOI:** 10.3390/pediatric13010013

**Published:** 2021-02-24

**Authors:** Désirée Caselli, Maurizio Aricò

**Affiliations:** Giovanni XXIII Children Hospital, Azienda Ospedaliero Universitaria Consorziale Policlinico, 70126 Bari, Italy; desiree.caselli@policlinico.ba.it

The Covid-19 pandemic is still raging. In the absence of effective therapies, while adopting masking and physical distancing, herd immunity remains the unmet need. Developing vaccines for SARS-CoV-2 immediately became an urgent need worldwide. In Russia, the Gam-COVID-Vac based on recombinant adenovirus type 26 (rAd26) and rAd type 5 (rAd5) has been developed [1,2] in China, a rAd5 vectored vaccine expressing the spike glycoprotein was developed [3,4]; furthermore, attempts to develop a vaccine are carried on in Cuba as well [5]. The decision of the European Union to pool the resources and to finance ex ante those groups who were in the main research pipeline, represented a completely novel approach and turned out to be a great success. It can also be considered the first really common effort of the EU affecting so deeply and so directly its citizens.

The RNA-based vaccine (Pfizer-BionTech and Moderna) is an absolute first-in-man but got to market under approval of the U.S. Food and Drug Administration (FDA) and the European Medicines Agency (EMA) in less than one year. Other types of vaccines, based on Adenovirus vector (AstraZeneca/Oxford University; Janssen) or on protein subunit (Novavax; Sanofi/GSK), are also rapidly progressing and becoming progressively available [6].

RNA-based vaccines are already in use in most European countries, and short-term data on efficacy are fully positive with a protection largely exceeding 90% starting from 1 week after administration of the boost dose, i.e., at 4 weeks from vaccination start, according to manufacturer’s guidelines [7,8].

Starting with V-Day on Christmas 2020, the European Union (EU) vaccination machinery has been working, with about 11 million doses distributed to European countries, including 3.1 million in Italy as of 15 February 2021, when the uptake of the first dose is 2.7% [9]. The target of herd immunity is in front of us, but still out of our reach [10]. To get there, we need the public health machinery (distribution, storage, selection of candidates, calls, vaccination and finally boosting, according to specific vaccine schedule) to all go on working smoothly. Difficulties concerning the regular flow of the vaccine doses have raised more than little worries [11] and, in some cases, initial delay in the speed of the entire process. Respect of the contracts between companies and the EU on timing and number of doses of vaccines delivered is pivotal in order to keep the pace of universal vaccination. Rumors on convenient re-assignment of aliquots of vaccines to other markets cannot be tolerated further; EU authorities must be able to reassure the EU population that every EU citizen will be protected regardless of individual income or country of origin, as they actually are doing. Delaying the administration of the expected second dose of RNA-based vaccine is also a matter of discussion today, with the only aim to buy time and room for expanding the number of subjects who have received at least one dose, while we work to make vaccine doses widely and timely available [12].

Beyond the above, the issue of considering SARS-CoV-2 vaccination as mandatory is approaching fast. If our aim is hampering the circulation of the virus, while re-opening social activities, which vaccination strategy may be more helpful, since universal coverage is not yet at hand? Vaccination of healthcare workers comes obvious to most of us. Their mandatory vaccination appears a possible, although still questionable and not straightforward, option in the near future. Are there other subgroups who might be considered candidates for enforced, if not mandatory vaccination? What about children? On 18 February, Klass et al. launched on the *New England Journal of Medicine* their proposal to treat COVID-19 as we have done for measles, and thus put the issue of mandatory vaccination on the table [13]. This will obviously relate to school activities. Schools have been locked down, for students of any age, for a very long time in most European countries, although not all of them [14]. Whether or not children should be vaccinated pertains to their role in the pandemic spread. Are they really harming fragile, senior relatives and citizens? Initial reports suggest that children transmit SARS-CoV-2 less easily than the influenza virus [15]. It would be uneasy to justify a decision to mandate vaccine in children for a disease that remains, up to date, far less frightening in most children than in adults or in seniors [16]; even more if the role of children in spreading the infection to adult and risk-subjects remains questionable. In a recent meta-analysis of the published literature on 213 household SARS-CoV-2 transmission clusters from 12 countries, only 3.8% transmission clusters were identified as having a pediatric index case. Asymptomatic index cases were associated with a lower secondary attack in contacts than symptomatic index cases (estimate risk ratio [RR], 0.17; 95% confidence interval [CI], 0.09–0.29). To determine the susceptibility of children to household infections, the secondary attack rate in pediatric household contacts was assessed and found to be lower than in adult household contacts (RR, 0.62; 95% CI, 0.42–0.91) [17].

In their review on the same topic, Opel et al. [18] a few months ago considered several crucial issues: the reproduction number (R_0_; i.e., the number of others to whom an infected person would spread the disease if placed in a totally susceptible population) is very high (around 15) for measles. With this background, approximately 92% to 94% of the population must be immune to prevent spread. This has been achieved by requiring two doses of measles vaccine for children in all US states before enrollment in school, with only very limited ways to opt out. Otherwise, R_0_ is much lower (around 1) for the influenza virus, and only a little higher, 2 to 2.5, for SARS-CoV-2 [19]. Moreover, Opel et al. suggested nine standard criteria potentially useful to guide whether a COVID-19 vaccine for children should be mandated [20,21]. These criteria address, obviously, safety of the vaccine and its efficacy; but more subtly, ask to demonstrate that vaccinating the infant, child, or adolescent may reduce the risk of pandemic spread especially among risk-subjects. It is noteworthy that some recent observations even point toward another direction [17,22,23,24].

These are the kind of data which are usually acquired through pre-marketing studies. In the case of the current COVID-19 pandemic, the time-frame did not allow the usual course of evaluation. Accumulation of the data needed to answer such questions might require years of research. Based on the above considerations, we have to preserve safety of vaccinations [25], not to expose this precious public health weapon to the queries of no-vax and negationists. The lesson of the now-discredited and retracted article suggesting a link between MMR vaccine and autism, published in the *Lancet* in 1998, caused a devastating attempt to development of universal vaccination campaigns. Anxiety has been deliberately exacerbated by antivaccine activists and organizations, despite extensive research that clearly showed no evidence of any verifiable link to neurodevelopmental disorders. Making SARS-CoV-2 vaccine mandatory for children today, in the absence of this information, might be unsafe and thus probably unwise.

## Data Availability

Not applicable.
